# Antimycobacterial Activity of *Sida hermaphrodita* (L.) Rusby (Malvaceae) Seed Extract

**DOI:** 10.3390/cells12030397

**Published:** 2023-01-22

**Authors:** Kinga Lewtak, Paulina Czaplewska, Jerzy Wydrych, Radosław Keller, Aldona Nowicka, Krzysztof Skrzypiec, Marta Julia Fiołka

**Affiliations:** 1 Department of Cell Biology, Institute of Biological Sciences, Maria Curie-Skłodowska University, Akademicka 19, 20-033 Lublin, Poland; 2 Laboratory of Mass Spectrometry, Intercollegiate Faculty of Biotechnology, University of Gdansk, Abrahama 58, 80-307 Gdansk, Poland; 3 Department of Functional Anatomy and Cytobiology, Institute of Biological Sciences, Maria Curie-Skłodowska University, Akademicka 19, 20-033 Lublin, Poland; 4 Analytical Laboratory, Institute of Chemical Sciences, Department of Chemistry, Maria Curie-Skłodowska University, Pl. M. Curie-Skłodowskiej 2, 20-031 Lublin, Poland; 5 Department of Immunobiology, Institute of Biological Sciences, Maria Curie-Skłodowska University, Akademicka 19, 20-033 Lublin, Poland

**Keywords:** *Mycobacterium smegmatis*, plant extract, antibacterial, plant antimicrobial peptides, *Sida hermaphrodita*, Virginia mallow, seeds

## Abstract

The current prevalence of such lifestyle diseases as mycobacteriosis and tuberculosis is a result of the growing resistance of microorganisms to the available antibiotics and their significant toxicity. Therefore, plants can successfully become a source of new therapeutic agents. The aim of this study was to investigate the effect of protein extract from *Sida hermaphrodita* seeds on the morphology, structure, and viability of *Mycobacterium smegmatis* and to carry out proteomic characterization of the protein extract. The analyses were carried out using fluorescence and transmission microscopy, atomic force microscopy, and spectroscopy. The proteomic studies were performed using liquid chromatography coupled to tandem mass spectrometry. The studies showed that the seed extract applied at concentrations of 50–150 µg/mL exerted a statistically significant effect on *M. smegmatis* cells, that is, a reduction of the viability of the bacteria and induction of changes in the structure of the mycobacterial cell wall. Additionally, the SEM analysis confirmed that the extract did not have a cytotoxic or cytopathic effect on fibroblast cells. The proteomic analysis revealed the presence of structural, storage, and enzymatic proteins and peptides in the extract, which are typical for seeds. Proteins and peptides with antimicrobial activity identified as vicillins and lipid-transporting proteins were also determined in the protein profile of the extract.

## 1. Introduction

Medicinal plants have been used as a major source of drugs for treatment and prevention of diseases for thousands of years. The current pharmaceutical industry has reached the present level owing to the use of plant-based traditional medicines [1]. Reports have revealed that more than half of all modern clinical drugs are of natural origin. Approximately 60% of anticancer compounds and 75% of drugs for infectious diseases are either natural products or their derivatives [2].

With their pharmacological properties, plant species of the *Sida* genus are widely used worldwide. *S. acuta*, *S. cordifolia*, *S. rhombifolia*, *S. cordata*, and *S. galheirensis* are the best-known phytopharmacological raw materials [3,4]. These species are a source of many compounds, for example, ephedrine, pseudoephedrine, and N-methyltryptophan, which are responsible for the antibacterial, analgesic, anti-inflammatory, hypoglycemic, antimalarial, and antioxidant properties of this plant material [5,6]. Antioxidant (27%) and antimicrobial (18%) activities are the most frequently mentioned pharmacological properties of *Sida* plants. Their leaves (46%) are the most common pharmaceutical raw material used for extraction of bioactive compounds, whereas their seeds account for 2%. To date, the only data on the antimicrobial activity of *S. hermaphrodita* seed extracts against *C. albicans* cells have been reported in our previous publication [7].

Virginia mallow (*Sida hermaphrodita*) is a tall perennial herb of the mallow family. Its potential economic importance is mainly related to the possibility of using the plant as a source of biomass for energy purposes, a raw material in the pulp and paper industry, fodder, a land reclamation tool in chemically degraded areas, and a source of honey [8,9]. In addition, the presence of mucous substances in Virginia mallow roots and leaves is comparable to the content of these components in a known pharmaceutical material, the marshmallow (*Althaea officinalis*), which may make Virginia mallow a valuable pharmaceutical raw material [10,11].

Mycobacteria are the etiological factors of dangerous infectious diseases, including human and animal tuberculosis. Non-tuberculosis mycobacteria other than the *Mycobacterium tuberculosis* complex and *Mycobacterium leprae* cause diseases called mycobacteriosis. The incidence of these diseases is increasing worldwide. Most often, mycobacteriosis causes lung infections. They also cause other ailments, for example, lesions of skin and soft tissues, and compromise the osteoarticular system and lymph nodes. Patients with comorbid respiratory diseases are particularly at risk of mycobacteriosis. Treatment of mycobacteriosis is difficult and requires long-lasting multi-drug therapy. Interestingly, the results of mycobacteriosis treatment are worse than the results of treatment of tuberculosis due to the higher frequency of the side effects of the antibiotics used. Therefore, there is a need for development of more effective therapies for mycobacteriosis. Additionally, the mechanisms leading to the onset and development of this disease should be elucidated in greater detail [12].

New therapies are being developed based on analogs of existing drugs or alternative therapeutics of natural origin. Over the past few years, progress has been made in the search for new natural tuberculostatics targeting specific *M. tuberculosis* strains. Secondary metabolites isolated from plants, bacteria, fungi, and aquatic organisms are their main sources [13].

Work with *M. tuberculosis* is difficult and requires highly professional conditions. The culture of the human pathogen requires special laboratory conditions with a high level of biosafety (level 3). Specialized training is required as well, as accidental infection may easily occur. *M. tuberculosis* is characterized by slow growth, and it takes two to three weeks for this strain to multiply. This makes experiments on this microorganism very time-consuming. Three species, that is, *Mycobacterium smegmatis*, *Mycobacterium bovis* (BCG strain), and *Mycobacterium marinum*, are the main models used for the analysis of *M. tuberculosis* [14]. The aim of the present research was to analyze the effect of protein extract obtained from S*. hermaphrodita* seeds on the viability and morphology of *M. smegmatis* cells. The extract has been analyzed biochemically and the characteristics of its antifungal activity have been described [7].

## 2. Materials and Methods

### 2.1. Microorganisms

The *Mycobacterium smegmatis* strain originated from the collection of microorganisms of the Department of Immunobiology, Maria Curie-Skłodowska University.

### 2.2. Plant Material

**Figure 1:**
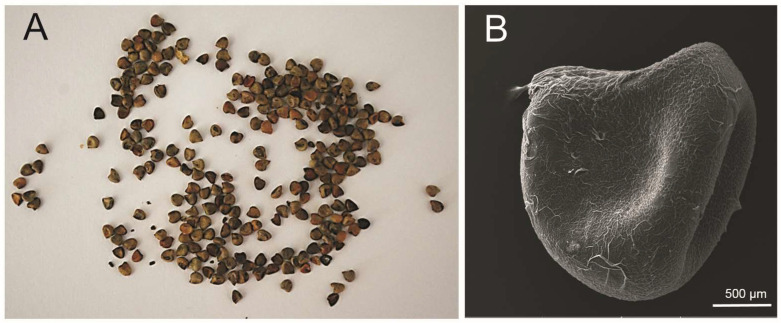
*S. hermaphrodita* seeds: (**A**)—macroscopic image; (**B**)—microscopic image obtained with a scanning electron microscope (SEM).

*S. hermaphrodita* seeds were purchased on a farm growing Virginia mallow commercially and distributing seeds. The material used for the research came from the 2011–2012 collections. Whole seeds (with the husk) were used to prepare the extract. The habit of the seeds is shown in Figure 1.

### 2.3. Preparation of Sida hermaphrodita Seed Extract

**Figure 2:**
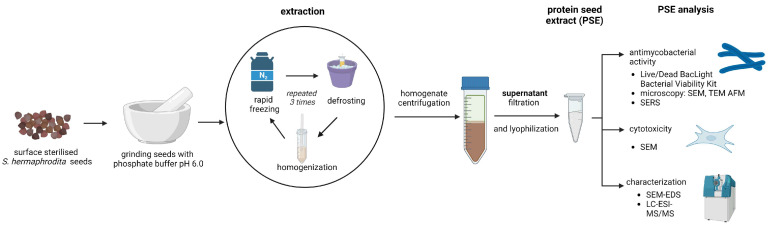
Scheme of seed extract preparation and analysis. Created with BioRender.com.

After surface sterilization in a 70% ethyl alcohol solution and rinsing with distilled water, the *S. hermaphrodita* seeds were subjected to mechanical homogenization in Sörensen buffer (pH 6.0). The homogenate was then disintegrated by snap-freezing in liquid nitrogen and thawing in an ice bath. The cycle was repeated three times. To obtain protein seed extract (PSE), the homogenate was centrifuged at 18,000× *g* for 10 min and filtered through a microbiological filter with a pore diameter of 0.22 µm (Millipore). A total of 10 g of plant material and 100 mL of phosphate buffer were used to prepare the extract. The concentration of protein in the extract was determined using the Bradford method [15]. The preparation of the extract and the experimental design are shown in Figure 2 (created with BioRender.com; the Academic License belongs to Paulina Czaplewska and the University of Gdańsk).

### 2.4. Preparation for Microscopy Techniques

*M. smegmatis* cells were treated with the *S. hermaphrodita* protein seed extract in liquid cultures. The samples were prepared by adding 6.25, 12.5, 18.75, 25, and 37.5 µL of the extract (to the final protein concentration of 25, 50, 75, 100, and 150 µg/mL, respectively) to 100 µL of the *M. smegmatis* mycobacterial suspension (OD600 = 0.5) and 100 µL of Sauton’s medium. The samples were supplemented to 250 µL with Sauton’s medium. A control sample with Sauton’s medium was prepared in parallel. The samples were incubated for 6 days at 37 °C with shaking (120 rpm).

Next, the cells of the control and experimental cultures were subjected to microscopic analysis. To prepare and decontaminate the mycobacterial cells for electron and atomic force microscopy, the NALC-NaOH solution-based procedure was used [16]. The mycobacterial cell suspension was centrifuged at 3000× *g* for 20 min. A total of 100 µL of a mixture composed of 1 mL of 1% N-acetyl-L-cysteine (Sigma) in 2.9% citric acid (Sigma) and 1.0 mL of 4% NaOH (Sigma) were added to the pellet and incubated at 37 °C for 20 min. Next, the cells were centrifuged again, and the resulting pellet was resuspended in 100 µL of phosphate buffer at pH 7.0 and used for further microscopic preparation.

### 2.5. Determination of Mycobacterium smegmatis Cell Viability

The LIVE/DEAD BacLight Bacterial Viability Kit allows quick determination of living and dead cells based on the integrity of their cell membranes [17]. Bacterial suspensions (10 µL) from the control and experimental groups were incubated with 3 µL of a dye mixture (5 µM Syto9 and 30 µM propidium iodide) for 15 min in the dark at room temperature. Then, 3 µL of the resulting suspension was applied to a microscope slide and observed under a Zeiss Axiovert confocal microscope with an LSM 5 Pascal scanning head.

The images of live and dead bacteria were collected as fluorescent green and red bacilli under a fluorescence microscope using appropriate single bandpass filter sets. The excitation/emission maxima were 480 nm/500 nm for Syto9 and 490 nm/635 nm for propidium iodide. The viability of the mycobacterial cells was estimated based on the percentage of Syto 9 fluorescence in the whole specimen. The images were analyzed using ImageJ 1.42q.

The minimum inhibitory concentration (MIC) for *M. smegmatis* was determined with the agar dilution method on solid Sauton’s medium (with 0.7% agar) [18]. The PSE with protein concentrations ranging from 6.25 to 150 μg/mL was analyzed. The MIC value corresponded to the lowest extract concentration at which no more than one colony developed. The experiment was repeated three times.

### 2.6. Scanning Electron Microscopy (SEM)

#### 2.6.1. Mycobacteria

After the treatment with the CSPE, the mycobacterial cells were fixed with 4% glutaraldehyde [19] in 0.1 M phosphate buffer, pH 7.0, and with 1% OsO_4_. Next, the cells were dehydrated in a graded acetone series (15%–100%), transferred to scanning electron microscope stages, and dried overnight in a desiccator. The dried cells were sputtered with gold using a Quorum Technologies Emitech K550X vacuum sputter-coater. The samples were analyzed using a Tescan VEGA3 LMU scanning electron microscope. The images were documented at a magnification of 20,000×.

#### 2.6.2. Fibroblasts

After incubation with the seed extract (experiment described by Lewtak et al. [7]), fibroblasts were fixed with 4% glutaraldehyde in 0.1 M phosphate buffer at pH 7.0. Next, the cells were mounted on slides, fixed with a 1% OsO_4_ solution for 1 h, and dehydrated in a series of acetone solutions (15%, 30%, 50%, 70%, 100%). Subsequently, the cells were dried in a desiccator using silica gel for 24 h and gold-coated with an Emitech K550X sputter coater (Quorum Technologies, Lewes, UK). The cells were analyzed using a Tescan Vega 3 scanning electron microscope (Tescan, Brno, Czech Republic).

### 2.7. Atomic Force Microscopy (AFM)

An atomic force microscope was used to visualize the surface of the mycobacterial cells after the treatment with the *S. hermaphrodita* seed extract. The analysis was performed using a NanoScope V atomic force microscope (Veeco Instruments Inc., Santa Barbara, CA, USA) equipped with a scanning probe. The measurements were made in the PeakForce QNM mode using the RTESPA probe. Three 2 µm × 2 µm fields were probed in each sample. The roughness was read from 10 fields with dimensions of 100 nm × 100 nm from each 2 μm × 2 μm image, and then the arithmetic mean was calculated from the results. Nanoscope Analysis version 1.4 (Veeco, USA) and WSxM version 5.0 (Nanotec, Madrid, Spain) programs were used to analyze the images.

### 2.8. Transmission Electron Microscopy (TEM)

The TEM analysis of PSE-treated mycobacteria was performed by negative staining [20]. After pretreatment with the NALC-NaOH solution, the *Mycobacterium* cells were fixed with 4% glutaraldehyde. After that, the bacterial cells were suspended in water mixed in a 1:1 ratio with a contrast solution (1% natrium silicotungstate, 0.5% ammonium molybdate) and micropipetted on TEM grids (3 mm mesh) coated with formvar film. The mixture was incubated for 10–15 min to pellet the cells on the mesh surface; afterwards, the solution was removed with filter paper. The grids were dried in a pre-vacuum during the specimen holder transfer into the microscope column. The observations were carried out using a Tecnai G2 T20 X-TWIN (FEI) transmission electron microscope.

### 2.9. Surface-Enhanced Raman Spectroscopy (SERS)

Surface-Enhanced Raman Spectroscopy (SERS) is a spectroscopic technique for measurement of Raman scattering radiation (inelastic light scattering) enhanced by depositing the analyzed substance on a metal surface or creating a metallic sol (by reducing the metal salt) with which the substance interacts. The SERS technique is widely used in intravital analyses of microbiological samples.

**Figure 3:**
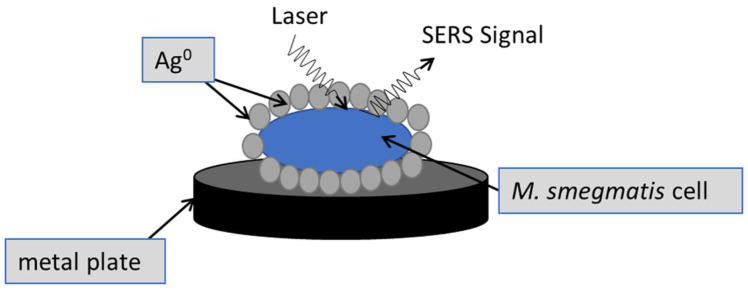
Diagram showing the formation of silver particles by chemical reduction and coating the *M. smegmatis* cells with Ag nanoparticles to enhance the Raman signal.

The analysis of *M. smegmatis* cells was based on the method consisting in coating the cells with silver (Ag) particles obtained by chemical reduction [21]. To prepare silver nanoparticle-coated cells, after the treatment with the protein seed extract, centrifugation (3000× *g*, 20 min), and washing with sterile water, the mycobacterial cells were resuspended in a 0.1 M sodium borohydride (NaBH_4_) aqueous solution. Next, the cells were washed, and the pellet was resuspended in a 0.05 M silver nitrate (AgNO_3_) aqueous solution. The samples were dried on metal plates and analyzed. Measurements were taken using an InVia Raman microscope from Renishaw (UK). A laser emitting light of 785 nm and 1 mW power was used for excitation. The spectra were processed (smoothed and baseline-corrected) using Renishaw’s WiRE 3.4 software (Renishaw, Wotton-under-Edge, UK). They were interpreted based on data published by Hamash and colleagues [22]. The scheme of the experiment is shown in Figure 3.

### 2.10. SEM-EDS Analysis of the Microstructure and Elemental Composition of S. hermaphrodita Seed Extract

The microscopic analysis of the structure and elemental microanalysis of the seed extract was performed using the SEM-EDS technique (Energy Dispersive X-ray Spectroscopy). The EDS microanalysis facilitates quantitative and qualitative identification of chemical elements contained in the analyzed material. The electrons from the beam interact with the atoms of the sample, resulting in an emission of X-rays, which are collected in the EDS detector, further processed, and presented in the form of a spectrum, that is, the dependence of the number of counts as a function of radiation energy. The value of the energy allows identification of elements in each sample, and the intensity of the peaks in the spectrum facilitates quantitative analysis. A sample of the freeze-dried and liquid extract was applied to the metal microscope stage and placed in the microscope chamber. The microstructure of the extract was imaged at a range of magnifications from 80 to 400,000×. The elemental composition analysis was carried out after excitation with a 10 kV beam. The observations and measurement were carried out using the high-resolution scanning electron–ion microscope Quanta 3D FEG (FEI).

### 2.11. LC-ESI-MS/MS Analysis of PSE

The LC-ESI-MS/MS (Liquid Chromatography coupled to tandem Mass Spectrometry) technique combines the resolution of high-performance liquid chromatography (HPLC) separation with the high mass accuracy of the mass spectrometer. In addition, the system uses electrospray ionization (ESI) as the sample ionization method.

To prepare the sample for the analysis, after reduction with dithiothreitol (Sigma-Aldrich, St Louis, MO, USA) and alkylation with iodoacetamide (Sigma-Aldrich, St Louis, MO, USA), digestion in a trypsin solution was applied according to the protocol described by Gundry and colleagues [23]. The samples were then separated using an Expert MicroLC 200 Plus System (Eksigent, Redwood City, CA, USA) on an Eksigent microLC ChromXP C18CL column (3 µm, 120 Å, 150 × 0.3 mm) in a 30 min gradient program. The program involved the use of two solvents: solvent A—0.1% formic acid solution in water and solvent B—0.1% formic acid solution in acetonitrile. The following elution scheme was used: (1) 0–2 min—10% solvent B, (2) 2–23 min—10–90% solvent B, (3) 23–28 min—90% solvent B, and (4) 28.1–30 min—10% solvent B. The eluate from the column was analyzed in the positive ion mode on a TripleTOF 5600+ hybrid mass spectrometer equipped with a DuoSpray Ion Source (SCIEX, Framingham, MA, USA). The microLC-MS/MS system was controlled by AB SCIEX Analyst TF 1.6 software. The experiments were performed in a data-dependent mode using parameters described by Lewandowska et al. [24]. The MS/MS spectra were analyzed based on the data available in the Malvales Uniprot protein database using ProteinPilot 4.5 software (SCIEX) and the Paragon algorithm with automatic false discovery rate analysis.

### 2.12. Statistical Analysis

The results obtained in the study are presented as the mean of at least three independent repetitions, taking into account the standard deviation (±SD). In the case of results meeting the assumptions of normality of distribution and equality of variance, one-way ANOVA was used for statistical data analysis. Statistical significance was assumed at * *p* < 0.05, ** *p* ≤ 0.01, and *** *p* ≤ 0.001. The analysis was performed using Statistica.

## 3. Results

### 3.1. Quantification of Mycobacterium Viability after Incubation with the S. hermaphrodita Extract

**Figure 4:**
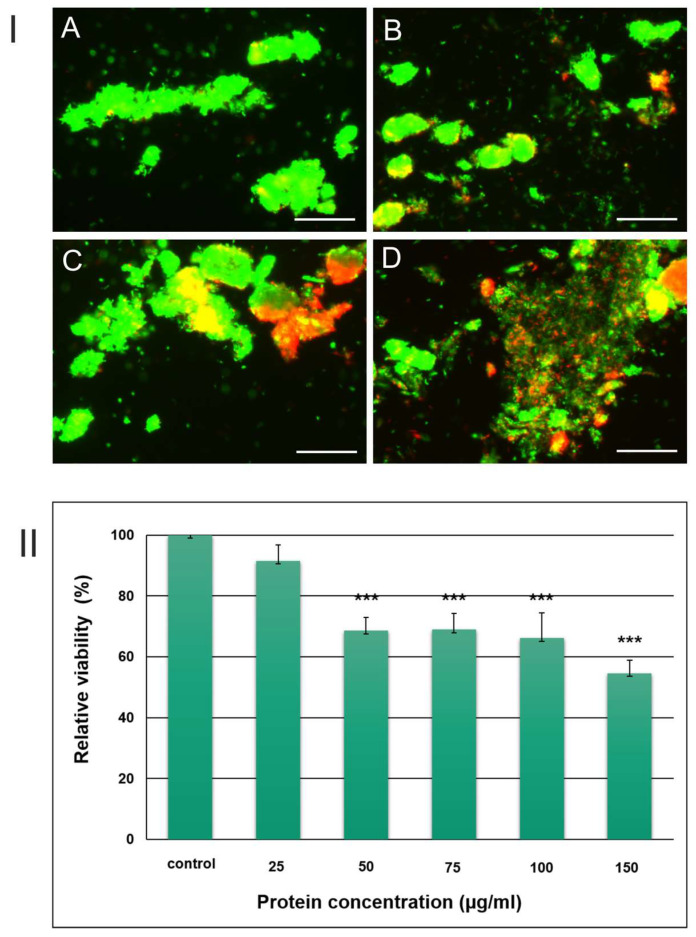
Viability of *M. smegmatis* cells in the control culture and cells incubated with the protein seed extract (PSE) for 6 days at 37 °C. (**I**). Visualization of *M. smagmatis* after PSE-treatment with LIVE/DEAD BacLight Bacterial Viability Kit: A—control culture, B—cells after treatment with PSE at the protein concentration of 25 µg/mL C—with PSE at 75 µg/mL; D—with PSE at 150 µg/mL. The scale bar corresponds to 50 µm. (**II**). Relative viability of *M*. *smegmatis* after treatment with PSE at different concentrations. The data are expressed as the mean with standard deviation (±SD) from three independent experiments. Statistical significance (compared to the control): *** *p* ≤ 0.001.

The survival rate of *M. smegmatis* cells after 6 days of incubation with the *S. hermaphrodita* protein seed extract was examined using the LIVE/DEAD BacLight Bacterial Viability Kit assay. Live bacterial cells emitted green fluorescence, while dead cells were red. The images shown are representative of the 20 micrographs taken (Figure 4I). The analysis of the results allowed us to determine the relative survival rate of *M. smegmatis* cells after the treatment with the protein extract of Virginia mallow seeds with protein concentrations of 25, 50, 75, 100, and 150 µg/mL. The values were 92%, 69%, 69%, 66%, and 55%, respectively. It was observed that cell viability decreased with the increase in the protein concentration in the extract sample. In the case of the extract with the concentrations of 50, 75, 100, and 150 µg/mL, the decrease in mycobacterial survival after 6 days of incubation (by 31%, 31%, 34%, and 45%, respectively) compared to the control was statistically significant. On the other hand, the statistical analysis of the results for the treatment with 25 µg/mL of the extract showed no significant changes in the viability of mycobacterial cells (Figure 4II). The MIC value of the *S. hermaphrodita* seed extract obtained with the agar dilution method was 25 µg/mL. The MIC value was confirmed by the LIVE/DEAD test.

### 3.2. SEM Analysis of M. smegmatis Cells after the Treatment with the S. hermaphrodita Extract

**Figure 5:**
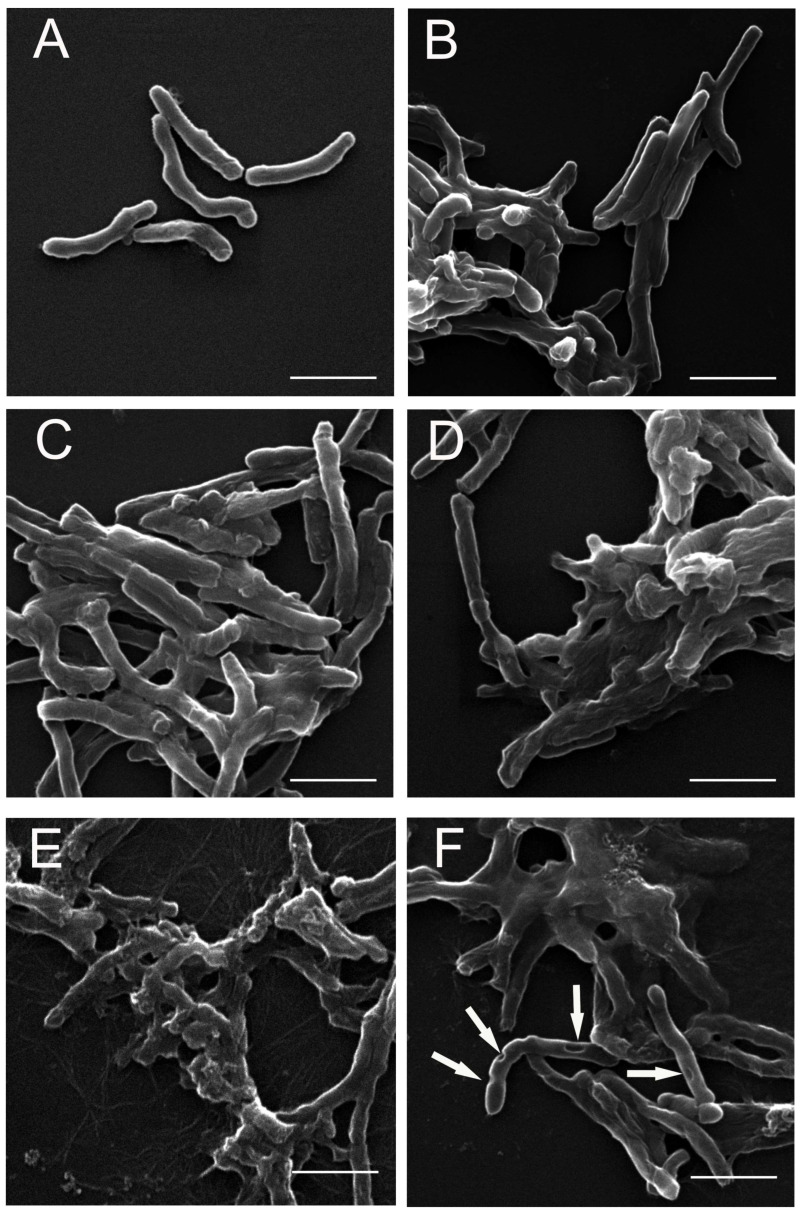
SEM microphotographs of *M. smegmatis* control cells and cells incubated with the *S. hermaphrodita* protein seed extract (PSE) for 6 days: (**A**)—*M. smegmatis* control culture cells; (**B**)—*M. smegmatis* cells after treatment with PSE at a concentration of 25 µg/mL; (**C**)—50 µg/mL; (**D**)—75 µg/mL, (**E**)—100 µg/mL; (**F**)—150 µg/mL. The surface of the extract-treated cells showed significant deformations and cavities. The arrows indicate defects in the continuity of the cell wall. The scale bar corresponds to 2 µm.

Scanning electron microscopy (SEM) was used to visualize the morphological changes in the *M. smegmatis* cells induced by the treatment with the protein extract of *S. hermaphrodita* seeds (PSE). After the microscopic analysis, representative photographs showing images of both samples: control mycobacterial cells and cells treated with the extract at the particular concentrations were selected. The results are shown in Figure 5.

The extract-untreated control *M. smegmatis* cells were characterized by a smooth cell wall surface, and their shape and size were typical (Figure 5A). In contrast, numerous cell deformations were observed in the mycobacterial cultures exposed to the extract at the concentrations of 25, 50, 75, 100, and 150 µg/mL. A general tendency of the cells after the incubation with the PSE was their merging into aggregates, within which deformed cells were visible (Figure 5B–F). The mycobacterial cells incubated with 100 and 150 µg/mL PSE were significantly shortened (Figure 5E) and exhibited an almost complete loss of structure, leading to the formation of amorphous cell aggregates (Figure 5F). In addition, in the case of cells incubated with the extract (150 µg/mL), there were defects in the continuity of the cell wall visible as indentations in the wall (Figure 5F).

### 3.3. AFM Analysis of M. smegmatis Cells after the Treatment with the S. hermaphrodita Extract

**Figure 6:**
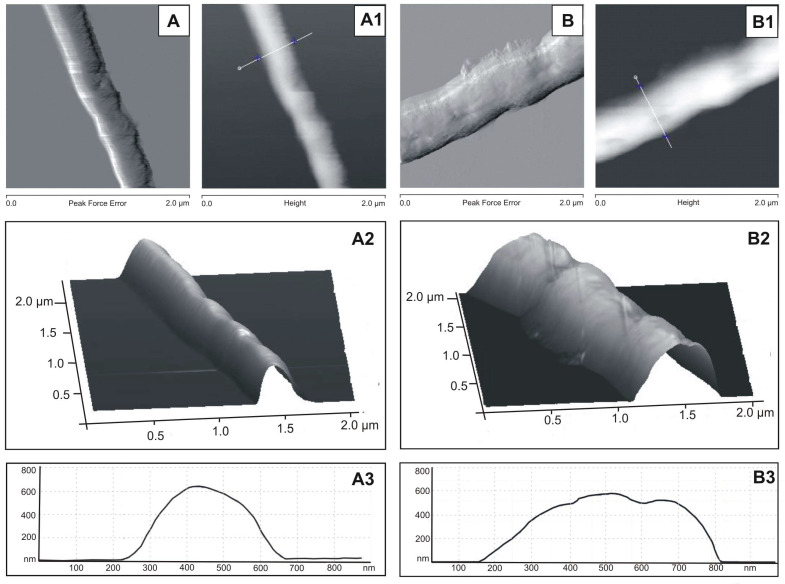
*M. smegmatis* cell surface imaged by AFM: (**A**–**A3**)—image of the control *M. smegmatis* cell surface; (**B**–**B3**)—image of the *M. smegmatis* cell surface after 6 days of incubation with 150 µg/mL of the *S. hermaphrodita* protein seed extract. (**A**,**B**)—general images of cells obtained in the Peak Force mode; (**A1**,**B1**)—images of cells obtained in the height mode; (**A2**,**B2**)—3D visualization of (**A**) and (**B**) images; (**A3**,**B3**)—profile of the mycobacterial cell surface; the profiles were made along the lines shown in the height images.

The analysis of mycobacteria using the atomic force microscopy (AFM) technique revealed that the surface of the *M. smegmatis* cells treated with the *S. hermaphrodita* seed extract at a concentration of 150 µg/mL was less rough than that of the control cells. In addition, the profile of the extract-treated cells differed from that of the control cells, that is, they were wider, more flattened, and irregular. Moreover, it was found that the average roughness of the extract-exposed cells was lower (Ra 19.4 nm 1.08) than the average roughness of the control cells (Ra 22.9 nm 1.75). Representative results of the AFM analysis are shown in Figure 6.

### 3.4. TEM Analysis of M. smegmatis Cells after the Treatment with the S. hermaphrodita Extract

**Figure 7:**
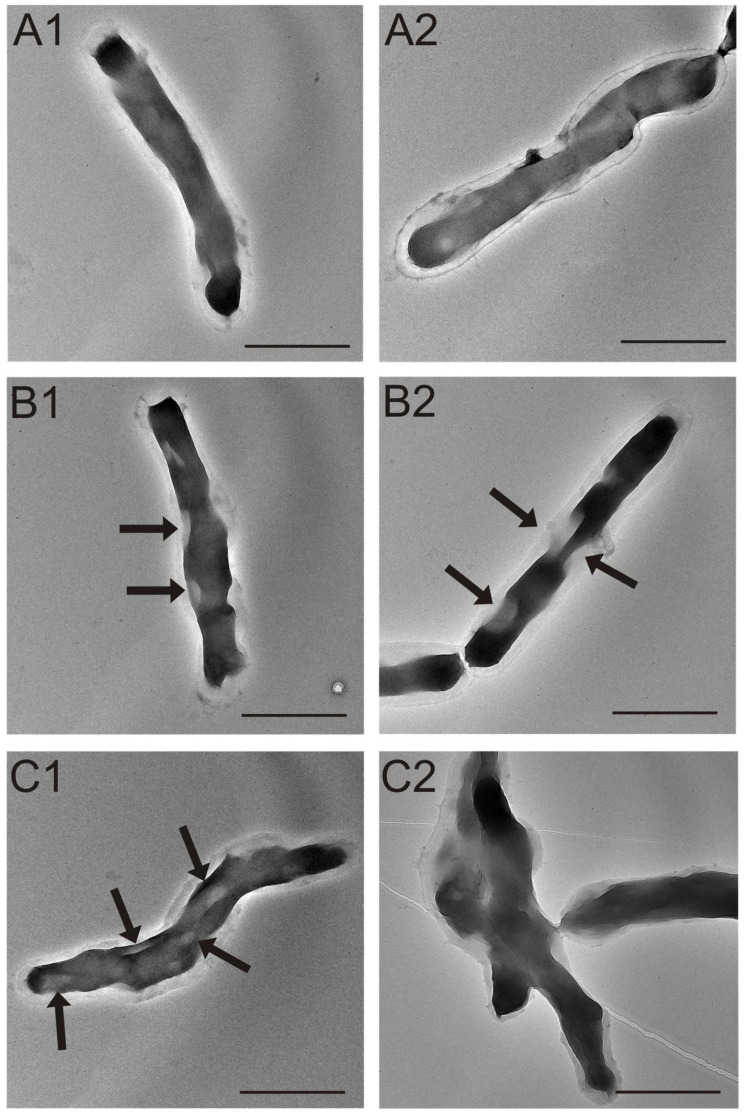
Transmission electron microscopy images of *M. smegmatis* cells: (**A1**,**A2**)—control *M. smegmatis* cells; (**B1**–**C2**)—cells treated with the *S. hermaphrodita* protein seed extract at a concentration of 150 µg/mL. Arrows indicate defects in mycobacterial cell wall continuity induced by the treatment with the extract. The scale bar corresponds to 2 µm.

The changes in the external structure of the *M. smegmatis* cell wall visualized by SEM and AFM were confirmed by transmission electron microscopy. Mycobacterial cells incubated with the *S. hermaphrodita* protein seed extract at the highest protein concentration (150 µg/mL) were used for these analyses. Representative images of the control and test samples are shown in Figure 7.

The control cells were characterized by a uniform cell wall and the shape and size typical of normal *M. smegmatis* cells (Figure 7A). In turn, numerous defects in the continuity of the cell wall were clearly visible on the surface of mycobacterial cells treated with the tested extract. These were indentations in the cell (the defects are marked with arrows in Figure 7B,C). In addition, the cells from the treated sample showed a tendency to aggregate, with simultaneous disappearance of the physiological shape (Figure 7D).

### 3.5. Analysis of Changes in the Chemical Composition of the M. smegmatis Cell Wall after Exposure to the S. hermaphrodita Extract Using Surface-Enhanced Raman Spectroscopy

**Figure 8:**
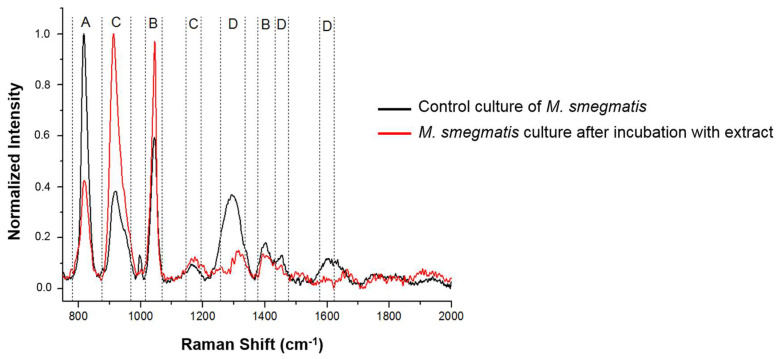
SERS spectra of cell wall surfaces of control *M. smegmatis* cells and cells incubated with the *S. hermaphrodita* protein seed extract at a concentration of 150 µg/mL for 6 days. The letters refer to signals characteristic of: A—nucleic acids, B—proteins, C—carbohydrates, D—lipids.

The next step of the study was intended to carry out analyses at the molecular level to supplement the microscopic images showing changes within the *M. smegmatis* cell wall after the incubation with the protein seed extract (PSE). To this end, the Surface-Enhanced Raman Spectroscopy (SERS) method was used. The analysis of the chemical composition of the cell walls using the SERS technique showed clear differences between the spectra of the control and treated cells (Figure 8). An increased number of carbohydrate- and protein-specific chemical bonds and a reduction in lipid chemical bonds were observed in the *M. smegmatis* cell wall after 6 days of exposure to the PSE with a protein concentration of 150 µg/mL, compared to the control culture. In the spectrum of the PSE-incubated mycobacteria, the peak corresponding to carbohydrates was more intense than in the spectrum of the control cells (C) (an increase in signal intensity at 1150 cm^−1^). The signals for nucleic acids and lipids were less intense (A and D). In contrast, the peak corresponding to proteins (B) was higher at 1050 cm^−1^ and slightly lower at 1400 cm^−1^. This indicates a quantitative change in the protein composition in the mycobacterial cell wall after exposure to the extract of Virginia mallow seeds.

### 3.6. Effect of the Seed Extract on Fibroblast Cells

**Figure 9:**
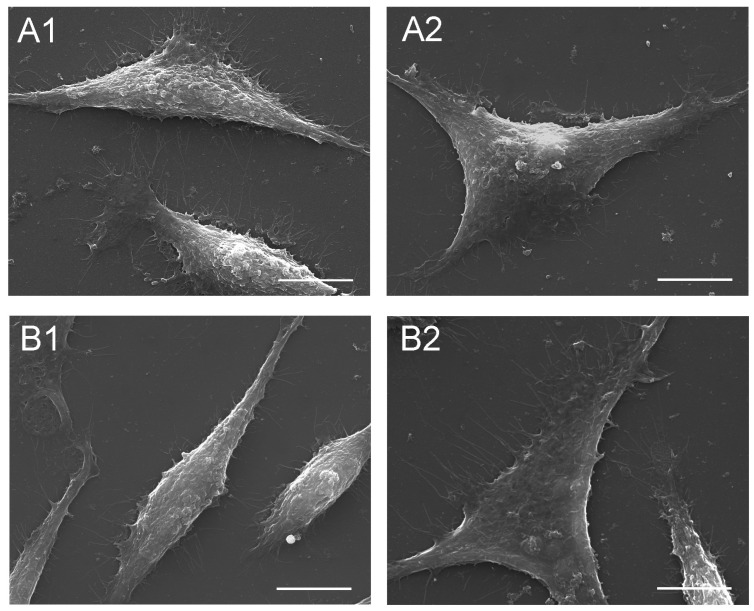
SEM imaging of fibroblasts: (**A1**,**A2**)—control fibroblasts; (**B1**,**B2**)—fibroblasts incubated with the *S. hermaphrodita* protein seed extract at a concentration of 100 µg/mL. The scale bar corresponds to 50 µm.

The absence of cytotoxic activity of the protein seed extract (PSE) was confirmed and described in a previous publication [7]. The SEM imaging confirmed the absence of changes in the general morphological picture and surface structure of the fibroblasts (Figure 9B1,B2) after incubation with PSE (at 100 µg/mL), compared to the control cells. Thus, the cells treated with the extract showed no cytopathic effect, compared to the control cells (Figure 9A1,A2).

### 3.7. Characterization of S. hermaphrodita Protein Seed Extract (PSE)

#### 3.7.1. Microanalysis of PSE Using Scanning Electron Microscopy and the X-ray Energy Dispersion Technique

The analysis of the microstructure and elemental composition of the Virginia mallow seed extract was carried out using a scanning electron microscope (SEM) with the X-ray dispersion technique (EDS). To this end, a sample of the extract was lyophilized and imaging was performed without prior preparation. The EDS microanalysis of the elemental composition of the extract showed the presence of elements characteristic of organic compounds, that is, C, N, O, Na, Mg, P, S, Cl, and Ca. The percentage content of individual elements is presented in Table 1.

**Figure 10:**
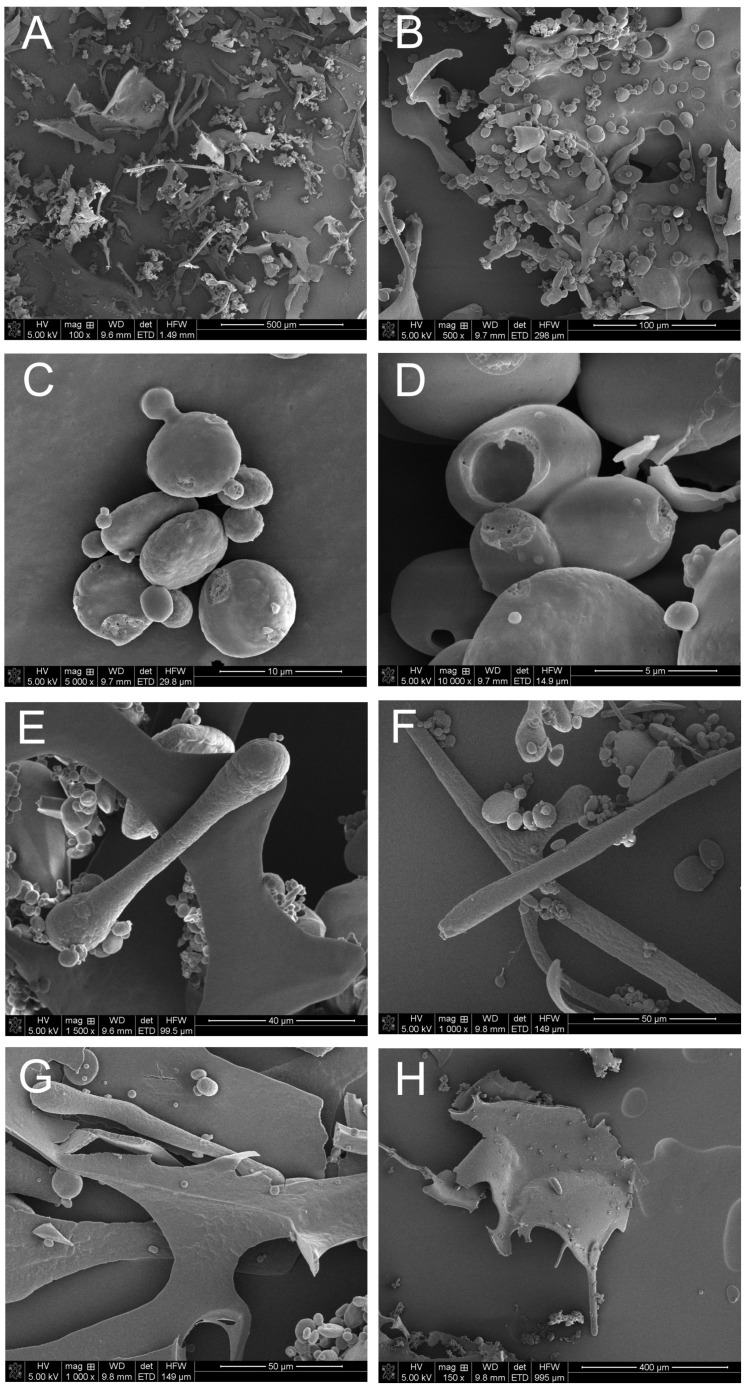
SEM micrographs of *S. hermaphrodita* seed extract: (**A**,**B**)—general image of the extract; (**C**,**D**)—spherical structures visible in the microscopic image of the extract; (**E**,**F**)—rod-shaped longitudinal structures; (**G**,**H**)—flat structures.

The analysis of the microstructure of the seed extract sample revealed an inhomogeneous structure of the preparation. The image was typical of a sample of organic origin being a mixture of many compounds (Figure 10A,B). The image showed spherical (Figure 10C,D), rod-shaped (Figure 10E,F), and flat structures (Figure 10G,H). The oval elements visible in images C and D are most likely starch grains.

#### 3.7.2. ESI LC MS Analysis of PSE

Classical proteomic analysis was performed to determine the protein composition of the protein seed extract in detail. Peptides obtained after digestion of the extract with trypsin were analyzed using ESI-LC-MS/MS, and the Malvales database (Uniprot) was used to identify these peptides. The proteomic analysis of the seed extract revealed the presence of structural, functional, and enzymatic proteins. The analysis of the results focused on groups of peptides identified as vicillins and lipid transfer proteins (LTPs) (Table 2). These peptides are mainly attributed storage and structural functions; however, they can also function as plant antimicrobial peptides.

## 4. Discussion

*M. smegmatis* is classified as an opportunistic pathogen and as mycobacteria other than *Mycobacterium tuberculosis* (MOTT) or non-tuberculous mycobacteria (NTM). Due to its rapid growth, non-pathogenicity, and the same unusual cell wall structure as in *M. tuberculosis*, *M. smegmatis* is often used as a model organism in preliminary screening studies [25,26,27,28]. *M. smegmatis* is a facultatively intracellular bacteria with the ability to survive inside macrophages, that is, the host’s immune cells. This contributes to their extreme effectiveness in infecting the host organism. A characteristic feature of mycobacteria is the specific composition of the cell wall, with over 60% of lipids. This feature provides these bacteria with high resistance to external factors, including drying, high and low temperature, and changes in pH [29,30].

There are many similarities between the saprophytic *M. smegmatis* strain and the pathogenic *M. tuberculosis* strain. The fundamental similarity is the biosynthesis of mycothiol, which is required for the production of the basic thiol necessary for the growth of *Mycobacterium* spp. [31]. *M. smegmatis* is a very convenient model for experiments because it is avirulent and easy to manipulate genetically. This species requires a laboratory only at biosafety level 1. No heavy infrastructure is required for working with pathogen species in this case. Therefore, this species was used as a model in the present research [32].

Plant extracts with anti-mycobacterial activity against both *M. tuberculosis* and saprophytic mycobacteria have been described in the literature. Three medicinal plants of the Lamiaceae family were shown to be highly active against mycobacterial strains [33]. Essential oils extracted with the standard method showed activity against *M. tuberculosis* H37Rv and against non-tuberculosis *M. kansasii* and *M. fortuitum* mycobacterial strains in the range of 39 to 156 µg/mL. Oils extracted from Lebanese plants *Micromeria barbata*, *Eucalyptus globulus*, and *Juniperus excelsa* exhibited essential antimycobacterial activity against selected *Mycobacterium* spp. strains: *M. tuberculosis*, *M. kansasii*, and *M. gordonae* [34]. Crude methanolic extracts from *Lantana camara* leaves, *Cryptolepis sanguinolenta* roots, and *Zanthoxylum leprieurii* stem barks showed antimycobacterial action against *M. smegmatis* (mc2155), as well as pan-sensitive (H37Rv) and rifampicin-resistant (TMC-331) *M. tuberculosis* [35].

It is undeniable that medicinal plants are an important source of new chemical substances with potential antimicrobial and therapeutic effects. Available data from the literature indicate that vegetative parts of plants are the most popular source of active compounds; however, seeds are increasingly being investigated by researchers. During germination, seeds must survive in a humid and pathogen-rich environment. As seeds absorb water, a significant amount of solutes is released into the surrounding environment, including sugars, organic acids, ions, amino acids, and proteins. Increased concentrations of these nutrients may lead to enhanced fungal and bacterial growth and cause pathogenic interactions. To counteract this problem, plant seeds have developed numerous defense mechanisms against pathogens [36], such as the synthesis and secretion of antimicrobial compounds, including proteins and peptides, which also determine the antimicrobial activity of seeds in vitro [37]. Studies have shown that the greatest number and variety of antimicrobial peptides is present in seeds [38].

The search for sources of anti-mycobacterial activity is a difficult challenge due to the resistance of the cell wall of these microorganisms. It is an insurmountable barrier for many antibiotic substances. Our analysis of extracts from a large group of plants has contributed to the isolation of an extract with high lysozyme-type activity. It is known that such activity is desirable for the digestion of the cell wall and is used in the professional preparation of specimens for diagnostic tests.

As reported by Barashkova and Rhogozhin [39], two groups of extractants are currently used to extract peptides from plant material. One is represented by aqueous solutions, including salt and acid solutions, buffer solutions, and water alone. The other group of extractants includes organic-based solutions, such as aqueous ethanol solutions. The most commonly used solutions for the extraction of proteins and peptides, including antimicrobial peptides (AMPs) of plant origin, are buffer solutions. Taking into account the ease and relatively low cost of extraction of desired antimicrobial substances, aqueous extracts can be an alternative method for producing medicinal substances, especially in the case of protein and peptide compounds.

The present analyses showed that the protein extract of *S. hermaphrodita* seeds applied at the lowest concentration (50 μg/mL) effectively reduced the survival rate of *M. smegmatis* cells by 31%. The greatest decrease in the cell survival rate (45%) was noted after the treatment with highest concentration of the extract (150 μg/mL). In addition, the SEM microscopic image of the seed extract-treated cells revealed numerous distortions in the mycobacterial cell wall, leading to the formation of amorphous cell aggregates. The SEM and TEM micrographs of the mycobacteria incubated with the seed extract at a concentration of 150 μg/mL showed cells with characteristic defects in the cell wall. As revealed by the atomic force microscopy (AFM) images, the mycobacteria subjected to the seed extract treatment had a flattened and less regular cell profile, compared to the control cells. The Surface-Enhanced Raman Spectroscopy (SERS) analysis of the *M. smegmatis* cells incubated with the Virginia mallow seed extract showed an increase in peak characteristics for carbohydrates and proteins and weaker signals for lipids in the mycobacterial cell wall. This is explainable, because the attachment of protein–polysaccharide compounds of the extract to the wall of mycobacteria caused the loss of its integrity and was associated with the loss of lipids in the wall. Earlier analyses of the extract indicated signals from sugars and proteins in the same regions on the electrophoretic gel, which most likely indicates the presence of protein–sugar complexes in the extract. The GC-MS analysis results revealed the presence of glucose and galactose in the extract [7].

The SEM microanalysis indicated the presence of starch in the preparation, which clearly confirms the presence of carbohydrates in PSE. Starch is a plant polysaccharide consisting of glucose subunits and acting as a reserve material. A comparable microscopic image of starch was presented by Odeku [40], Miranda et al. [41], Kaur et al. [42] and Leemud et al. [43].

Valuable information on the proteomic composition of the protein seed extract (PSE) was provided by the ESI-LC-MS/MS analysis. It revealed the presence of a number of functional, structural, and storage proteins characteristic of seeds. Some of these proteins and peptides were identified as vicillins or vicillin-like peptides and lipid transfer proteins (LTPs). These two types of peptides can act as both storage proteins and antimicrobial peptides in seeds. Vicillins are oligomers with a molecular mass from 150 to 170 kDa. They serve mostly as storage proteins of seeds, especially in legumes, and constitute as much as 70–80% of the amount of total protein in seeds of these plants [35,36]. In turn, plant LTPs (lipid transfer proteins) are small cysteine-rich proteins with a molecular weight in the range of 7–9 kDa. LTPs have been shown to be able to bind and transport lipids (phosphatidylinositol, phosphatidylcholine, and galactolipids) and other small molecules, indicating their broad substrate specificity. Some LTPs are classified as antimicrobial peptides with antifungal and antibacterial activity [44,45]. Antimicrobial peptides can form peptide–lipid complexes, resulting in changes in wall thickness, pore formation, changes in curvature, and modification of cell membrane electrostatics [46]. A similar effect was caused by a glycolipid–peptide complex isolated from the extracellular metabolites of *Raoultella ornithinolytica* bacteria, that is, an intestinal symbiont of the earthworm *Dendrobaena veneta*. After incubation of *M. smegmatis* with the active complex, loss of cell wall integrity and nanomechanical changes in the surface of mycobacterial cells were observed [47].

Our previous research on *S. hermaphrodita* seed extract showed effective antifungal activity against the clinical strain of *C. albicans* with no cytotoxic or cytopathic effect on human skin fibroblasts, which is an important feature in the evaluation of a new antimicrobial formulation [7]. A new application of the extract was submitted for protection and was granted a patent by the Patent Office in Poland [48].

Mainstream medicine is becoming increasingly receptive to the use of antimicrobial and other drugs derived from plants, as traditional antibiotics are becoming ineffective. The use of natural products derived from medicinal plants as an alternative source to combat infections in humans may also be beneficial due to their lower costs and lesser toxicity [49]. The analysis of plant material with anti-mycobacterial activity leads to the exploration of natural bioactive substances that may contribute to the creation of new pharmaceuticals to combat mycobacteriosis.

Summing up the present research, *S. hermaphrodita* seed extract is a promising preparation for the production of an anti-mycobacterial drug because it not only exerts a significant effect destroying mycobacterial cells, but also does not show a cytotoxic effect in relation to normal human fibroblasts. Given the data characterizing the preparation in chemical terms, the next step will involve isolation of an active agent, most likely a complex composed of both protein and sugar compounds, and assessment of its endotoxicity, as is performed in the case of pharmaceutical preparations. Only then will it be possible to plan in vivo experiments using laboratory animals. Currently, since medicine based on synthetic preparations is not sufficiently effective in treatment, the production of pharmaceuticals is increasingly often based on plant-origin preparations that have not been tested so far. In the event of a shortage of anti-mycobacterial antibiotics with limited toxicity, *S. hermaphrodita* seed extract has the potential to be used as a new pharmaceutical of plant origin.

## Figures and Tables

**Table 1:** Elemental composition of *S. hermaphrodita* seed extract determined after excitation of the preparation with a 10 kV beam. Wt%—weight percentage of a given element in the sample; At%—atomic percentage of a given element in the sample. The results presented are the mean of three independent measurements.

Element	Wt%	At%
**C**	48.40 (±2.45)	56.15 (±1.74)
**N**	15.47 (±2.01)	15.39 (±1.55)
**O**	27.47 (±1.98)	23.93 (±0.90)
**Na**	0.58 (±0.04)	0.35 (±0.02)
**Mg**	5.39 (±0.15)	3.09 (±0.24)
**P**	1.03 (±0.01)	0.46 (±0.02)
**S**	0.50 (±0.03)	0.22 (±0.008)
**Cl**	0.23 (±0.005)	0.09 (±0.003)
**Ca**	0.92 (±0.01)	0.32 (±0.019)
**Total**	100.00	100.00

**Table 2:** Proteins and peptides identified as vicillins and lipid transfer proteins present in the PSE (according to the Malvales database, Uniprot). The table takes into account the parameters characterizing the proteins and peptides: the number of identified peptides (95% confidence) and the percentage of peptide sequence coverage.

N.	Name	Accession	Species	Peptides (95%)	% Cov
1.	uncharacterized protein LOC107929052 similar to Vicilin GC72-A GOSHI	A0A1U8LMX7	GOSHI	47	29.1
2.	vicilin-like antimicrobial peptides 2-2	A0A0B0PUP3	GOSAR	31	44.9
3.	vicilin-like seed storage protein At2g28490	A0A1U8P4G8	GOSHI	28	26.8
4.	vicilin-like seed storage protein At2g18540	A0A1U8K3C6	GOSHI	8	24.2
5.	vicilin GC72-A	A0A1U8LQ34	GOSHI	35	34.0
6.	non-specific lipid-transfer protein	U5HUL1	GOSRA	1	22.5
7.	uncharacterized protein similar to non-specific lipid-transfer protein-like protein At5g64080 GOSHI	A0A0D2SK78	GOSRA	2	10.5
8	non-specific lipid-transfer protein-like protein At5g64080	A0A1U8MVT2	GOSHI	1	18.9

## Data Availability

The data presented in this study are available on request from the corresponding author.

## References

[1] Pan L., de Blanco E.J.C., Kinghorn A.D., Osbourn A., Lanzotti V. (2009). Plant-derived natural products as leads for drug discovery. Plant—Derived Natural Products.

[2] McChesney J.D., Venkataraman S.K., Henri J.T. (2007). Plant natural products: Back to the future or into extinction?. Phytochemistry.

[3] Medeiros I.A., Santos M.R.V., Nascimento N.M.S., Duarte J.C. (2006). Cardiovascular effects of *Sida cordifolia* leaves extract in rats. Fitoterapia.

[4] Rodrigues F.C., de Oliveira A.F.M. (2020). The genus *Sida* L. (Malvaceae): An update of its ethnomedicinal use, pharmacology and phytochemistry. S. Afr. J. Bot..

[5] Ahmad I., Mehmood Z., Mohammad F. (1998). Screening of some Indian medicinal plants for their antimicrobial properties. J. Ethnopharmacol..

[6] Sutradhar R.K., Rahman A.K.M.M., Ahmad M., Bachar S.C., Saha A. (2006). Analgesic and anti-inflammatory principle from *Sida cordifolia* Linn. J. Biol. Sci..

[7] Lewtak K., Fiołka M.J., Czaplewska P., Macur K., Kaczyński Z., Buchwald T., Szczuka E., Rzymowska J. (2019). *Sida hermaphrodita* seeds as the source of anti—*Candida albicans* activity. Sci. Rep..

[8] Denisiuk W. (2005). Possibilities of using Virginia mallow in the power engineering. Agric. Eng..

[9] Nahm M., Morhart C. (2018). Virginia mallow (*Sida hermaphrodita* (L.) Rusby) as perennial multipurpose crop: Biomass yields, energetic valorization, utilization potentials, and management perspectives. GCB Bioenergy.

[10] Wolski T., Styk B. (1989). Sida hermaphrodita—A new herbal plant. Herb. News.

[11] Borkowska H., Wardzińska K. (1999). Influence of sowing quantity and harvest dates on diversification of Virginia mallow biomass yields. Ann. UMCS Sec. E Agric..

[12] Sharma S.K., Upadhyay V. (2020). Epidemiology, diagnosis & treatment of non-tuberculous mycobacterial diseases. Indian J. Med. Res..

[13] Nowakowska J., Ciura K., Rudnicka-Litka K. (2016). Natural anti-tuberculosis drugs. Adv. Phytother..

[14] Shiloh M.U., Champion P.A. (2010). To catch a killer. What can mycobacterial models teach us about *Mycobacterium tuberculosis* pathogenesis?. Curr. Opin. Microbiol..

[15] Bradford M.M. (1976). A rapid and sensitive method for the quantitation of microgram quantities of protein utilizing the principle of protein-dye binding. Anal. Biochem..

[16] Morcillo N., Imperiale B., Palomino J.C. (2008). New simple decontamination method improves microscopic detection and culture of mycobacteria in clinical practice. Infect. Drug Resist..

[17] Lahiri R., Randhawa B., Krahenbuhl J. (2005). Application of viability-staining method for *Mycobacterium leprae* derived from the athymic (nu/nu) mouse foot pad. J. Med. Microbiol..

[18] Sirgel F.A., Wiid I.J., van Helden P.D., Parish T., Brown A. (2009). Measuring minimum inhibitory concentrations in mycobacteria. Mycobacteria Protocols. Methods in Molecular Biology.

[19] Verma J., Rohilla A., Khuller G.K. (1999). Alterations in macromolecular composition and cell wall integrity by ciprofloxacin in *Mycobacterium smegmatis*. Lett. Appl. Microbiol..

[20] Harris J.R. (2007). Negative staining of thinly spread biological samples. Methods Mol. Biol..

[21] Efrima S., Zeiri L. (2009). Understanding SERS of bacteria. J. Raman Spectrosc..

[22] Hamasha K., Sahana M.B., Jani C., Nyayapathy S., Kang C.M., Rehse S.J. (2010). The effect of Wag31 phosphorylation on the cells and the cell envelope fraction of wild-type and conditional mutants of *Mycobacterium smegmatis* studied by visible-wavelength Raman spectroscopy. Biochem. Biophys. Res. Commun..

[23] Gundry R.L., White M.Y., Murray C.I., Kane L.A., Fu Q., Stanley B.A., Van Eyk J.E. (2009). Preparation of proteins and peptides for mass spectrometry analysis in a bottom-up proteomics workflow. Curr. Protoc. Mol. Biol..

[24] Lewandowska A.E., Macur K., Czaplewska P., Liss J., Łukaszuk K., Ołdziej S. (2017). Qualitative and quantitative analysis of proteome and peptidome of human follicular fluid using multiple samples from single donor with LC-MS and SWATH methodology. J. Proteome Res..

[25] Cordone A., Audrain B., Calabrese I., Euphrasie D., Reyrat J.M. (2011). Characterization of a *Mycobacterium smegmatis* uvrA mutant impaired in dormancy induced by hypoxia and low carbon concentration. BMC Microbiol..

[26] Lelovic N., Mitachi K., Yang J., Lemieux M.R., Ji Y., Kurosu M. (2020). Application of *Mycobacterium smegmatis* as a surrogate to evaluate drug leads against *Mycobacterium tuberculosis*. J. Antibiot..

[27] Andries K., Verhasselt P., Guillemont J., Göhlmann H.W., Neefs J.M., Winkler H., Van Gestel J., Timmerman P., Zhu M., Lee E. (2005). A diarylquinoline drug active on the ATP synthase of *Mycobacterium tuberculosis*. Science.

[28] Chaturvedi V., Dwivedi N., Tripathi R.P., Sinha S. (2007). Evaluation of *Mycobacterium smegmatis* as a possible surrogate screen for selecting molecules active against multi-drug resistant *Mycobacterium tuberculosis*. J. Gen. Appl. Microbiol..

[29] Pfyffer G.E., Jorgensen J.H., Carroll K.C., Funke G., Pfaller M.A., Landry M.L., Richter S.S., Warnock D.W. (2015). *Mycobacterium*: General characteristics, laboratory detection, and staining procedures. Manual of Clinical Microbiology.

[30] Bagieńska M., Rzewuska M. (2010). Occurrence of mycobacteria from the *Mycobacterium tuberculosis* complex in animals—Transmission of selected species between humans and animals. Vet. Life.

[31] Sundarsingh T.J.A., Ranjitha J., Rajan A., Shankar V. (2020). Features of the biochemistry of *Mycobacterium smegmatis*, as a possible model for *Mycobacterium tuberculosis*. J. Infect. Public Health.

[32] Li X., Mei H., Chen F., Tang Q., Yu Z., Cao X., Andongma B.T., Chou S.H., He J. (2017). Transcriptome Landscape of *Mycobacterium smegmatis*. Front. Microbiol..

[33] Kazemian H., Heidari H., Yamchi J.K., Zandi H., Taji A., Yazdani F., Hamzehloo G., Ghanavati R., Rahdar H.A., Feizabadi M.M. (2018). In vitro anti-mycobacterial activity of three medicinal plants of *Lamiaceae* family. Recent Pat. Antiinfect. Drug Discov..

[34] El Omari K., Hamze M., Alwan S., Osman M., Jama C., Chihib N.E. (2019). In-vitro evaluation of the antibacterial activity of the essential oils of *Micromeria barbata*, *Eucalyptus globulus* and *Juniperus excelsa* against strains of *Mycobacterium tuberculosis* (including MDR), *Mycobacterium kansasii* and *Mycobacterium gordonae*. J. Infect. Public Health.

[35] Tuyiringire N., Mugisha I.T., Tusubira D., Munyampundu J.P., Muvunyi C.M., Vander Y. (2022). In vitro antimycobacterial activity of medicinal plants *Lantana camara*, *Cryptolepis sanguinolenta*, and *Zanthoxylum leprieurii*. J. Clin. Tuberc. Other Mycobact. Dis..

[36] Marcus J.P., Green J.L., Goulter K.C., Manners J.M. (1999). A family of antimicrobial peptides is produced by processing of a 7S globulin protein in *Macadamia integrifolia* kernels. Plant J..

[37] Vieira Bard G.C., Nascimento V.V., Oliveira A.E., Rodrigues R., Da Cunha M., Dias G.B., Vasconcelos I.M., Carvalho A.O., Gomes V.M. (2014). Vicilin-like peptides from *Capsicum baccatum* L. seeds are α-amylase inhibitors and exhibit antifungal activity against important yeasts in medical mycology. Biopolymers.

[38] Finkina E.I., Melnikova D.N., Bogdanov I.V., Ovchinnikova T.V. (2019). Peptides of the innate immune system of plants. Part I. Structure, biological activity, and mechanisms of action. Russ. J. Bioorg. Chem..

[39] Barashkova A.S., Rogozhin E.A. (2020). Isolation of antimicrobial peptides from different plant sources: Does a general extraction method exist?. Plant Methods.

[40] Odeku O. (2013). Potentials of tropical starches as pharmaceutical excipients: A review. Starch/Stärke.

[41] Miranda J., de Carvalho L.M.J., Castro A.V.I. (2019). Scanning electron microscopy and crystallinity of starches granules from cowpea, black and carioca beans in raw and cooked forms. Food Sci. Technol..

[42] Kaur H., Gill B.S., Karwasra B.L. (2018). In vitro digestibility, pasting, and structural properties of starches from different cereals. Int. J. Food Prop..

[43] Leemud P., Karrila S., Kaewmanee T., Karrila T. (2020). Functional and physicochemical properties of Durian seed flour blended with cassava starch. J. Food Meas. Charact..

[44] Bard G.C.V., Zottich U., Souza T.A.M., Ribeiro S.F.F., Dias G.B., Pireda S., Da Cunha M., Rodrigues R., Pereira L.S., Machado O.L. (2016). Purification, biochemical characterization, and antimicrobial activity of a new lipid transfer protein from *Coffea canephora* seeds. Genet. Mol. Res..

[45] McLaughlin J.E., Darwish N.I., Garcia-Sanchez J., Tyagi N., Trick H.N., McCormick S., Dill-Macky R., Tumer N.E. (2021). A lipid transfer protein has antifungal and antioxidant activity and suppresses fusarium head blight disease and DON accumulation in transgenic wheat. Phytopathology.

[46] Abedinzadeh M., Gaeini M., Sardari S. (2015). Natural antimicrobial peptides against *Mycobacterium tuberculosis*. J. Antimicrob. Chemother..

[47] Fiołka M.J., Grzywnowicz K., Mendyk E., Zagaja M., Szewczyk R., Rawski M., Keller R., Rzymowska J., Wydrych J. (2015). Antimycobacterial action of a new glycolipid-peptide complex obtained from extracellular metabolites of *Raoultella ornithinolytica*. APMIS.

[48] Maria Curie-Skłodowska University (2019). Virginia Mallow (*Sida hermaphrodita*) Seed Extract for Use in Combating Infections Caused by the *Candida albicans* Fungus. Patent PL.

[49] Sher A. (2009). Antimicrobial activity of natural products from medicinal plants. Gomal J. Med. Sci..

